# The feasibility of implementing high-intensity interval training in cardiac rehabilitation settings: a retrospective analysis

**DOI:** 10.1186/s13102-020-00186-9

**Published:** 2020-06-29

**Authors:** Kimberley L. Way, Sol Vidal-Almela, Marja-Leena Keast, Harleen Hans, Andrew L. Pipe, Jennifer L. Reed

**Affiliations:** 1 Exercise Physiology and Cardiovascular Health Lab, Division of Cardiac Prevention and Rehabilitation, University of Ottawa Heart Institute, Ottawa, Canada; 2 School of Human Kinetics, Faculty of Health Sciences, University of Ottawa, Ottawa, Canada; 3 Institut du Savoir Montfort, Hôpital Montfort, Ottawa, Canada; 4 Faculty of Medicine, University of Ottawa, Ottawa, Canada

**Keywords:** Cardiovascular disease, Exercise, High-intensity interval training, Feasibility, Adherence, Safety

## Abstract

**Background:**

Cardiovascular disease is the leading cause of death worldwide. Notwithstanding the well-known benefits of cardiac rehabilitation (CR), adherence to CR remains low, particularly in women. High-intensity interval training (HIIT) has received specific attention as an emerging exercise-training paradigm that addresses frequently cited barriers to CR (i.e. lack of motivation/enjoyment and time, perceiving exercise regime as tiring/boring) and improves cardiovascular risk factors. Previous studies have examined the safety of HIIT in CR; there is little evidence on the feasibility of HIIT in CR. The aims of this study were to evaluate the feasibility of HIIT within a CR setting and examine the sex differences regarding the feasibility of such programming.

**Methods:**

Patients attended an on-site HIIT CR program (10-min warm-up, 25 min of interspersed high-intensity [HI - 4 min at 85–95% HRpeak] and lower intensity [LO - 3 min at 60–70% HRpeak] intervals, 10-min cool-down) twice weekly for 10 weeks. Heart rate (HR) and the Borg rating of perceived exertion (RPE) scale (6–20 points) were recorded at each session. Feasibility was assessed by: [1] attendance and compliance: the number of sessions attended and the compliance to the prescribed HI and LO HR ranges; [2] the patient experience: patients’ perceived effort, program difficulty, if the program was challenging and satisfying; and, [3] safety. Descriptive statistics were used to report the means and their variations. Mann-Whitney U tests and Chi-square analyses were performed to examine sex-differences.

**Results:**

A total of 151 patients (33% women, 57.5 ± 9.1 years) attended the HIIT program and completed 16 ± 5 classes with a low attrition rate (11.3%). Most patients met or exceeded the prescribed target HR for the HI (80%) and LO (84%) intervals, respectively. Patients reported a “somewhat hard” RPE for HI (14 ± 2 points) and “very light” for LO (10 ± 2 points) intervals. All patients were satisfied with the program and found it challenging. Most patients found HIIT to be difficult (7 ± 2 points, scale range 0–10 points), yet safe (97%). Three vasovagal episodes occurred and more women dropped-out of the program than men (*p* < 0.01).

**Conclusions:**

HIIT is a feasible, safe and well-received exercise paradigm in a CR setting.

## Background

Cardiovascular disease (CVD) is the leading cause of death worldwide [1]. Following a cardiovascular event, participation in exercise-based cardiac rehabilitation (CR) is recommended; such programs improve functional capacity, enhance psychological health and reduce cardiovascular mortality [2]. Despite the well-known benefits of participation in CR, adherence rates are low, particularly among women [3]. Patients report a number of barriers to participating in traditional CR including poor self-efficacy, low motivation, and time constraints [4]. Given the low attendance rate in traditional CR programs (mean: 66 ± 18%, range: 37–85% session attendance) [5], there is a need to examine the feasibility of other innovative exercise programs within CR settings.

There is growing interest in implementing high-intensity interval training (HIIT) in CR settings given the significant cardiovascular health improvements observed in adults with coronary artery disease [6–9] and heart failure [10, 11] when compared to traditional CR (moderate-to-vigorous intensity continuous aerobic exercise for 30–60 min) [12]. HIIT consists of repeated bouts of high-intensity exercise interspersed with lower intensity active/passive periods of recovery [13]. HIIT is an appropriate exercise paradigm for CR settings; current American, Canadian and European CR guidelines [12] recommend the prescription and progression of moderate-to-vigorous intensity continuous aerobic exercise. Despite evidence that suggests HIIT is safe for adults with CVD [14, 15], concerns remain regarding the feasibility of HIIT in CR [16]. This can be examined by assessing the attendance, compliance, and experience of patients and monitoring adverse events (safety) to determine if such programs are appropriate for ‘real world’ settings [17]. The majority of clinical trials to date, while providing brief statements on the attendance, compliance, drop-out rates and adverse events experienced with HIIT [7, 8, 18, 19], have inadequately reported these parameters. Some investigators, for instance, simply reported the mean intensity at which individuals exercised during HIIT [8, 18] while others noted only the compliance to the high- and low-intensity work bouts [20, 21]. No studies to date have comprehensively evaluated the patient’s perspective on the experience, satisfaction and safety with HIIT. Clinicians, as a consequence, lack the information which would allow them to determine the feasibility of introducing such programs to their patients. It is clear that more detailed reporting on the adherence to HIIT in a CR setting is needed.

Women frequently attend fewer CR exercise classes than men (mean difference in the ratio of sessions attended to those prescribed: − 3.4, 95% confidence interval: − 6.9 to − 0.3%, *p* = 0.03) [5]; it is important to determine which exercise programs may be most appealing to women. To date, studies that have examined the effects of HIIT on cardiovascular health in adults with CVD have involved predominantly men [22]. A recent women-only HIIT study by Reed et al. demonstrated greater clinically meaningful mental health improvements with HIIT when compared to moderate-to-vigorous intensity continuous exercise [2]. Further, Terada et al. found that women in CR experienced greater reductions in anxiety severity (as assessed by the Hospital Anxiety and Depression Scale) with HIIT (− 1.7 ± 2.7 vs. -0.4 ± 2.8 points, *p* = 0.036) when compared to men; yet men had larger improvements with moderate-to-vigorous intensity continuous exercise [23]. This provides promising evidence for the use of HIIT for women and underscores the importance of formally examining the impact of such programs for cardiac patients.

The principal purpose of this study was to conduct an exploratory retrospective analysis of the feasibility (as defined by the attendance, compliance, patient experience and safety) of a HIIT program in patients attending CR. The secondary purpose was to explore sex differences regarding the feasibility of such a program in CR.

## Methods

### Study design

This was a retrospective mixed-methods analysis to evaluate the feasibility of HIIT in a CR setting. Ethics approval for this study was obtained from the Ottawa Health Sciences Network Research Ethics Board (Protocol #: 20170721-01H). This study was conducted in line with the Declaration of Helsinki.

### Participants

Patients were referred by a physician or nurse practitioner in the community or using an automatic referral process at the University of Ottawa Heart Institute (UOHI), to an on-site CR program at the UOHI between January 2014 and May 2019. Eligible participants were those who: (i) had CVD (e.g. coronary artery disease, arrhythmias, valvular disease, stroke or transient ischemic attack, spontaneous coronary artery dissection, or heart failure) or CVD risk factors; (ii) had a baseline exercise level ≥ 4 metabolic equivalents (METS [≥ walking pace of 4.0 mph]); (iii) did not have contraindications for participating in high-intensity exercise; (iv) were able to sustain at least 30 min of aerobic exercise; and, (v) were able to independently self-monitor and report heart rate and rating of perceived exertion responses during exercise sessions.

### High-intensity interval training

Participants attended a group-based HIIT class led by a CR Physiotherapist (MLK) twice weekly for 10 weeks in the Cardiac Prevention and Rehabilitation Centre at the UOHI. The 45-min classes followed a modified Norwegian HIIT protocol [10] which consisted of: (i) a 10-min warm-up at 60–70% peak heart rate (HR_peak_); (ii) 4 × 4-min of high-intensity intervals (HI) at 85–95% HR_peak_ interspersed with 3 min of lower intensity intervals (LO) at 60–70% HR_peak_; and, (iii) 10-min cool-down at 60–70% HR_peak_ with resistance and stretching exercises. Peak HR was determined by a graded exercise test (GXT). In cases where GXT data were not available, HR_peak_ was estimated using the Gellish formula: 207-(0.70 x age) [24]. For those taking β-blockers, 30 bpm were subtracted from their estimated HR_peak_ to address the HR blunting effect of these medications [24].

Patients were provided the option to complete HIIT using: (i) aerobic exercise equipment (treadmill, cycle ergometer, elliptical, etc.) or (ii) dance/movement-based routines. All participants, regardless of exercise choice, completed the HIIT with musical accompaniment of a tempo appropriate for high or lower intensity training. The initial two weeks of the 10-week program were designed to allow the participants to familiarize themselves with the HIIT protocol. All participants monitored their exercise HRs by wearing a chest strap which displayed HR values through Polar HR monitors (Polar RS800CX, Polar Electro Oy, Kempele, Finland) or on aerobic exercise equipment. For patients using Polar HR monitors, values were displayed on a television in front of the participants in the Polar Team iPad application. Each of the participant’s HR recordings were verified by the supervising physiotherapist.

Participants were instructed to keep their HR within the appropriate target training range (i.e. 85–95% HR_peak_ or 60–70% HR_peak_ dependent on the interval), and to adjust movement or workload to stay within these exercise-intensity target ranges. HR was recorded after the first and last HI and LO intervals at each session. Participants were also encouraged to attain a Rating of Perceived Exertion (RPE) (6–20 scale) [25] of 15–17 (“hard to very hard”) during the HI and 11–13 (“light to somewhat hard”) during the LO intervals. Patients were instructed to record a typical RPE representing the effort of all the HI and LO intervals during HIIT. At the end of each session, participants received a 5-min educational talk addressing the self-management of CVD (e.g. physical activity, diet, medications and stress-management).

### Outcome measures

#### Feasibility

To assess the feasibility of HIIT in CR, exercise attendance, compliance, the patient experience and safety were examined.

##### Exercise attendance and compliance

Exercise attendance was assessed by the number of classes participants attended. High attendance to the CR program was defined as being present at ≥70% of the classes based on a previous protocol paper examining the feasibility of HIIT in CR [17]. Exercise compliance was assessed as the ability to complete the prescribed intensity for the HI and LO intervals. The HRs across all classes for the HI and LO intervals for each patient were averaged and compared to their target HR prescription. For instance, where patients exercised below, within or above the prescribed HR ranges, these were coded as “does not comply”, “complies” and “exceeds”. As patients were also encouraged to aim for a target HI and LO RPE range, we assessed the compliance to these ranges using the same approach as the HR data.

##### Patient experience

Participants were asked, upon program completion, to complete a feedback questionnaire which comprised of 20 questions regarding their experience with HIIT. The questionnaire was developed by scientists and clinicians involved in CR at the UOHI. For the purposes of this study, we analyzed questions regarding exercise intensity using a 10-point Likert scale with “0” being “not difficult at all” to “10” being “extremely difficult”. Patients were asked if HIIT was challenging using a Yes or No question, and whether patients were satisfied with the program that was offered using a Yes or No question. To further assess exercise intensity, RPEs across all classes for the HI and LO intervals, respectively, were averaged.

##### Safety

Safety was assessed by enumerating reported adverse events during the study period and the response to a single question regarding the participant’s perceptions of the safety of the program using a Yes or No question.

### Qualitative data

The feedback questionnaire also comprised open-ended questions regarding the HIIT program. The questions that were analysed for themes associated with the attendance, compliance, patient experience and safety were: (i) “Satisfied with the program and would recommend to others”; (ii) “Favourite part of the program”; and, (iii): “Additional comments or concerns”. Data analysis was undertaken using an inductive thematic analysis approach. This involved identifying repeated comments/experiences that were described by patients and coding these responses to determine themes.

### Estimated cardiorespiratory fitness

A symptom-limited peak GXT on a treadmill using an individualized ramp protocol (i.e. treadmill stress test) was completed at baseline and following the CR program by cardiac stress technologists in the Department of Cardiac Imaging at the UOHI. The ramp protocol involves walking or jogging at a constant speed (e.g. 2.0, 3.0, or 4.0 mph) dependent on participants’ functional abilities with a 1.7% increase in grade every minute until volitional fatigue is achieved. HR was measured throughout the test using an electrocardiogram. Estimated peak exercise capacity (V̇O_2_peak) was calculated using the ACSM Walking equation which takes into consideration the speed and grade reached in the final stage of the test: V̇O_2_ peak (mL/kg/min) = Final speed (m/min) × 0.1 + final grade x final speed (m/min) × 1.8 + 3.5.

### Participant characteristics

Research assistants extracted demographic and clinical information from the CR clinical database including age, ethnicity, marital status, education, smoking status, medication use and cardiovascular diagnoses.

### Anthropometry

Height was measured to the nearest 0.5 cm, body mass was measured to the nearest 0.1 kg, and body mass index (BMI) was calculated (kg/m^2^). Waist circumference was measured to the nearest 0.5 cm at the midpoint between the lower costal margin and iliac crest while participants stood with arms at their sides, feet 25–30 cm apart and abdomen relaxed.

### Resting blood pressure and heart rate

Resting blood pressure and HR were measured using an automated blood pressure monitor (Bp-TRU, Canada; or, Welch Allyn, Canada) by CR staff at baseline and following the CR program. These measures followed standardized procedures [24].

### Statistical analysis

Analyses were performed using SPSS for Windows (Version 26; IBM Corp, Armonk, NY, USA). All outcome variables were tested for normality using Shapiro-Wilk tests. For feasibility outcomes, descriptive statistics were used to describe the attendance, compliance, patient experience and safety. To assess sex-differences in participant characteristics and feasibility outcomes, independent t-tests and Mann-Whitney U tests were used for continuous variables for normally distributed and non-normally distributed data, respectively. Chi-square tests were used for categorical variables. A sensitivity analysis was performed to ascertain where differences in compliance to target HR ranges existed between participants whose HR_peak_ was determined by a GXT or the Gellish equation. Data are reported as means ± standard deviations, unless otherwise noted, and *p* < 0.05 was considered statistically significant.

## Results

### Participants

Descriptive data for the participants is shown in Table 1. Most (> 70%) participants were Caucasian, married and non-smokers. On average, participants were overweight, normotensive (due to medical management), with a ‘high-risk’ waist circumference (≥ 90 cm) for cardiometabolic diseases [26]. Most (> 50%) were taking anti-platelets, β-blockers, anti-dyslipidemics and angiotensin-converting enzyme (ACE) inhibitors. Of the patients who participated in HIIT, 67 patients (31% women, 69% men) completed the feedback questionnaire.

**Table 1:** Participant Characteristics

	Total ***N*** = 151	Women ***N*** = 50	Men ***N*** = 101	***P*** value (sex difference)
**Demographics, mean ± SD / n (proportion[%])**				
Age (years)	57.4 ± 9.5	57.5 ± 9.1	57.3 ± 9.7	0.981
Sex (% men)	101 (67)	–	–	–
Ethnicity (% Caucasian)	127 (87)	40 (85)	87 (88)	0.732
Marital status (% married)	112 (76)	31 (66)	81 (81)	0.177
Education (% four years College/University)^a^	68 (47)	19 (41)	49 (49)	0.195
Smoker (%)^a^	9 (6)	2 (4)	7 (7)	0.782
**Physical Measures, mean ± SD**				
Height (cm)^a^	171.0 ± 9.3	164.5 ± 8.7	174.6 ± 7.6	0.000**
Body mass (kg)	82.3 ± 16.9	77.0 ± 18.1	85.3 ± 15.5	0.006**
BMI (kg/m^2^)^a^	28.0 ± 4.9	28.0 ± 6.1	28.0 ± 4.3	0.279
Waist circumference (cm)^a^	97.5 ± 12.3	94.2 ± 14.7	99.1 ± 10.6	0.037*
Resting systolic blood pressure (mmHg)	123 ± 16	125 ± 17	122 ± 15	0.291
Resting diastolic blood pressure (mmHg)	75 ± 10	75.3 ± 8.7	74 ± 10	0.542
Resting heart rate (bpm)	65 ± 12	68 ± 14	63 ± 10	0.008**
V̇O_2peak_ (mL/kg/min)^a^	32.5 ± 7.1	28.9 ± 7.1	32.5 ± 7.1	0.000**
**Medications, n (proportion [%])**				
Anti-platelets	142 (94)	44 (88)	98 (97)	0.060^b^
β-blockers	109 (72)	31 (62)	78 (77)	0.049*
Anti-dyslipidemics	134 (84)	32 (60)	102 (95)	0.000**
ACE inhibitors	83 (55)	25 (50)	58 (57)	0.388
Angiotensin-receptor blockers	11 (7)	4 (8)	7 (7)	1.000^b^
Calcium channel blockers	20 (13)	7 (14)	13 (13)	0.847
Anti-coagulants	15 (10)	6 (12)	9 (9)	1.000^b^
Anti-depressants	16 (11)	8 (16)	8 (8)	0.091^b^
Anti-diabetics	12 (8)	4 (8)	8 (8)	1.000^b^
Anxiolytics	4 (3)	1 (2)	3 (3)	1.000^b^
**Cardiovascular History n (proportion [%])**				
Coronary artery disease	113 (75)	29 (58)	84 (83)	0.002**^c^
Angina	18 (12)	9 (18)	9 (9)	0.105
Arrhythmias	19 (13)	7 (14)	12 (12)	0.712
Ablation	2 (2)	1 (2)	1 (1)	1.000^b^
Valvular disease	19 (13)	7 (14)	12 (12)	0.712
Stroke/TIA	3 (2)	1 (2)	2 (2)	1.000^b^
SCAD	1 (1)	1 (2)	0 (0)	0.331^b^
Heart Failure	1 (1)	1 (2)	0 (0)	0.331^b^
PCI	89 (59)	24 (48)	65 (64)	0.055
CABG	27 (17)	1 (2)	25 (25)	0.000**
PCI + CABG	7 (5)	0 (0)	7 (7)	0.096^a^
Primary Prevention	5 (3)	4 (8)	1 (1)	0.041*^b^

*Abbreviations*: *ACE* angiotensin-converting enzyme, *BMI* body mass index, *CABG* coronary artery bypass graft, *PCI* percutaneous coronary intervention, *SCAD* spontaneous coronary artery dissection, *TIA* tranisent ischemic attack; V̇O_2peak_, peak exercise capacity. * Significant difference between sexes (p < 0.05). **Significant difference between sexes (p < 0.01). ^a^ Missing data. ^b^ Fisher’s Exact test in instances where > 20% of cells had an expected count of < 5. ^c^ Continuity correction in instances where > 20% of cells had an expected count of < 5. Values are presented as means ± standard deviations or frequency (%)

Men were taller, had greater body mass, waist circumference, V̇O_2peak_ and lower resting HR than women (*p* < 0.05). Further, more men were taking β-blockers and anti-dyslipidemics when compared to women (p < 0.05). More men suffered from coronary artery disease and had undergone a coronary artery bypass graft surgery (p < 0.05). Significantly more women participated in CR for the primary prevention of CVD than men. There were no other significant differences in demographics, anthropometrics, physical measures, medication use or cardiovascular conditions observed between men and women (*p* > 0.05).

### Feasibility outcomes

#### Exercise attendance and compliance

**Fig. 1:**
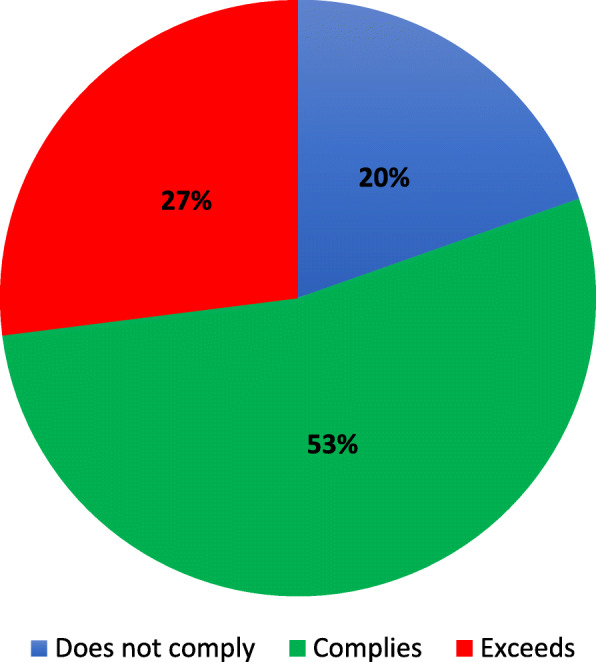
Compliance to the high-intensity intervals of the high-intensity interval training protocol. “Does not comply” refers to a mean HR during classes < 85–95% HR_peak_; “Complies” refers to a mean HR during classes with 85–95% HR_peak;_ “Exceeds” refers to a mean HR  > 95% HR_peak_

**Fig. 2:**
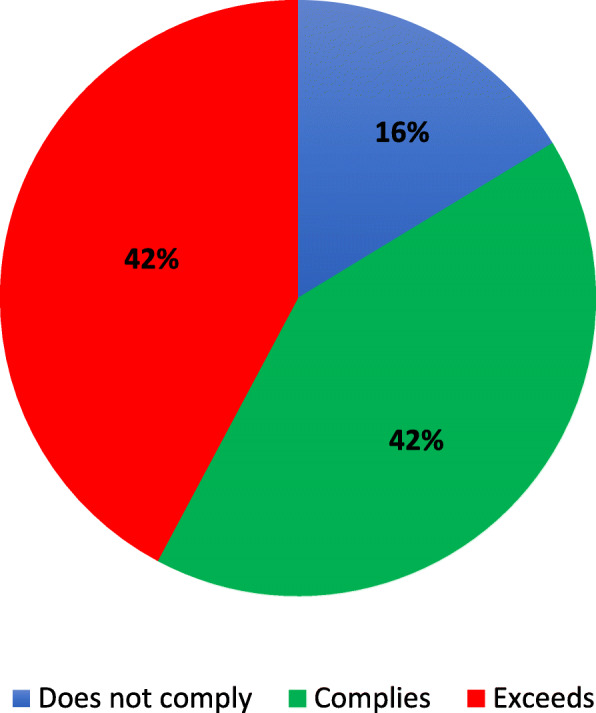
Compliance to the lower intensity intervals of the high-intensity interval training protocol. “Does not comply” refers to a mean HR during classes < 60–70% HR_peak_; “Complies” refers to a mean HR during classes with 60–70% HR_peak;_ “Exceeds” refers to a mean HR > 70% HR_peak_

On average, participants attended 16 ± 5 HIIT classes (out of 20 classes), with most patients (73%) completing ≥70% of the classes. Most participants were able to meet or exceed (HI: 80.4%, LO: 83.7%) the target HR ranges for the HI and LO intervals (Figs. 1 and 2). For the target RPE ranges, most participants reported exercising at an RPE lower than the encouraged HI (58%) and LO ranges (61%). The sensitivity analysis revealed no significant difference between the GXT (*n* = 129) or the Gellish equation (*n* = 11) methods for patients’ compliance to their target HR ranges (HI and LO, *p* > 0.05). While there was a low attrition rate (11.3%), significantly more women dropped-out of the program than men (*p* < 0.05, Table 2). No sex differences were found for class attendance and compliance with the exercise prescription (p > 0.05).

**Table 2:** Feasibility Outcomes

	All ***N*** = 151	Women ***N*** = 50	Men ***N*** = 101	P value (sex differences)
**Attendance and Compliance**				
Classes Attended^a^	16 ± 5	15 ± 6	16 ± 5	0.106
Attended ≥70% of classes^a^	103 (73)	34 (69)	69 (75)	0.474
Dropouts^a^	17 (11)	10 (20)	7 (7)	0.034*^b^
*Compliance to HI HR*^a^				0.931
Does not comply	28 (20)	10 (19)	18 (20)	
Complies	76 (53)	25 (52)	51 (54)	
Exceeds	36 (27)	13 (29)	23 (26)	
*Compliance to HI RPE*^a^				0.287
Does not comply	88 (58)	23 (58)	65 (66)	
Complies	43 (29)	16 (40)	27 (28)	
Exceeds	–	–	–	
*Compliance to LO HR*^a^				0.825
Does not comply	24 (16)	9 (17)	15 (16)	
Complies	58 (42)	21 (42)	37 (41)	
Exceeds	57 (42)	18 (40)	39 (43)	
*Compliance to LO RPE*^a^				0.571
Does not comply	83 (61)	25 (66)	58 (59)	
Complies	43 (32)	12 (30)	31 (32)	
Exceeds	3 (2)	–	3 (2)	
**Patient Experience**				
RPE HI^a^	14 ± 2	14 ± 2	14 ± 1	0.729
RPE LO^a^	10 ± 2	10 ± 2	10 ± 2	0.330
Difficulty of Class (0–10)^a^	7 ± 2	7 ± 1	7 ± 2	0.547
Challenging (Y/N)^a^	60 (100)	19 (100)	41 (100)	–
Satisfied with HIIT (Y/N)^a^	64 (100)	16 (100)	48 (100)	–
Safe (Y/N)^a^	62 (97)	14 (88)	48 (100)	0.060^c^
**Exercise Prescription**^a^				
GXT	129 (89)	–	–	–
Gellish Equation	11 (8)	–	–	–
Sensitivity analysis – HI HR				0.074
Sensitivity analysis – LO HR				0.910

*Abbreviations*: *GXT* graded exercise test, *HI* high-intensity, *HIIT* high-intensity interval training, *HR* heart rate, *LO* lower-intensity, *RPE* rating of perceived exertion, *Y/N* yes/no. *Significant difference between sexes (p < 0.05). ^a^ Missing data. ^b^ Continuity correction in instances where > 20% of cells had an expected count < 10.^c^ Fisher’s Exact test in instances where > 20% of cells had an expected count < 5. Values are presented as means ± standard deviations or frequency (%)

The qualitative data revealed that attending HIIT classes was difficult if an individual did not live near the UOHI or have transportation to the classes (Table 3): E.g. *“If I lived closer and had someone to get me there, I would not hesitate to attend and fully complete the program.”* Patients suggested that a local program may have improved their attendance: E.g. *“Local program, such as in Cornwall or Alexandria would be great! I live near Alexandria, so it is about 1 1/2 hour drive to The Heart Institute. Please let me know if there are local cardiac programs.”*

**Table 3:** Qualitative Analysis

Feasibility Outcomes	Common Themes
**Higher Attendance and Compliance**	Close Location for Classes
**Positive Patient Experience**	High Program Satisfaction Increased Confidence in Ability to Exercise Increased Social Interactions Enjoyment from High-Intensity Exercise
**Increased Patient Safety**	Supervision and Support from Staff Understanding Physical Capabilities and Limits Access to HR Monitoring

*Abbreviations*: *HR* heart rate

#### Patient experience

On average, patients’ perceived exertion during the HI intervals as “somewhat hard” (RPE: 14 ± 2) and “very light” (RPE: 10 ± 2) during the LO intervals. Most patients found the intensity of the HIIT class difficult (7 ± 2 [scale range: 0 to 10]). All patients found HIIT challenging and were satisfied with the program. No sex differences were found for any patient experience outcomes (i.e. perceived exertion, intensity difficulty, challenging program, program satisfaction [*p* > 0.05]).

The qualitative data showed that patients were satisfied with HIIT and most patients would recommend the program to other people: E.g. *“Absolutely! I would recommend to anybody. It was a great way to exercise and have fun at the same time.” “I was very well satisfied with the program. I would most certainly recommend it highly to others.”*

While no patients were dissatisfied with the program, some provided constructive feedback on how to improve the experience: E.g. *“A couple of the tunes don’t have a well-defined beat when starting - hard to find the start of the 8 count.” “(The scheduled class time) A little challenging because of work and dog care.” “The ambient noise sometimes covers the instructor’s voice.”*

Some patients found the HI intervals (*“the high-intensity portions”*) and *“getting the heart rate up and working hard with others”* was their favourite part of the program.

The social aspect of the HIIT classes was a common theme amongst patient responses which enhanced their experience: E.g. *“Looking forward to seeing the instructors and everyone at the centre.” “The people - honestly I’ll miss the routine of seeing everyone 2/week.” “Being part of a group is like being part of a team. You must do it and that is good. Necessary!”*

Patients also reported they were more confident in their ability to exercise: E.g. *“Gaining confidence in being able to move around/exercise/get my HR up. This was achieved by being pushed and urged to work out harder.” “I came into the program feeling very insecure with my AFIB diagnosis, not knowing how much I could do in terms of returning to exercise and my confidence has been restored … I’m back at my gym.”*

#### Safety

Three vasovagal episodes were reported during the HIIT program. No delayed adverse events were reported. Ninety-seven percent of patients reported the program to be ‘safe’ at all times.

The qualitative data showed that patients felt safe during classes because of the supervision and support they received from staff: E.g. *“I couldn’t feel safer - In fact I wish I could stay!” “The personal attention given to each person and the real concern everyone had with us.” “The program provides individual treatment and personalized care for each patient.”*

Patients also felt HIIT was within their physical capabilities and “*knowing my (their) capabilities and limits”*: E.g. *“It was a good way to improve my fitness level but still have the confidence that it was tailored to my circumstances.” “The ease of acceptance of your physical abilities at the beginning, very motivating + motivated instructors.” “The empathy and support from leaders was top shelf. They also pushed me to push my heart into vigorous territory which I wouldn’t have done without their expertise.”*

Further, HR monitoring was another aspect of safety that patients appreciated: E.g. *“Heart monitor was very helpful.” “Really like the heart rate/% display on screen. Very helpful in reinforcing awareness of exertion levels.”*

## Discussion

This study is the first, to our knowledge, to provide a comprehensive evaluation of the feasibility of HIIT in CR settings. In this retrospective analysis, we found that most patients attended ≥70% of the scheduled HIIT classes and were able to exercise at the prescribed HI (85–95% HR_peak_) and LO (60–70% HR_peak_) target HR ranges. Yet, most patients reported lower RPE values than the encouraged target HI (15–17 points) and LO (11–13 points) ranges for the HR targets prescribed. Interestingly, most patients found the HIIT program difficult and classes challenging. Yet, all participants reported that they were satisfied with HIIT. Adverse events were rare (0.0013% occurrence) and the majority of patients perceived the classes to be safe. Our sex-based comparisons revealed that more women dropped-out of the HIIT program than men. For all other outcomes, there were no significant sex differences. Our findings show that HIIT appears to be a feasible and well-tolerated exercise paradigm for patients undergoing CR.

While there have been some investigations demonstrating low attendance with HIIT [8, 20], most studies have reported high attendance (≥ 70% of the scheduled sessions) with HIIT in cardiac patients [7, 18, 19, 21, 27]. Specifically, Moholdt and colleagues revealed that individuals in a HIIT program (4 × 4 min at 85–95% HR_max_ with 3-min active recoveries at 70% HR_max_, two supervised sessions and one home session per week) attended 57% of the CR classes offered across a 12-week intervention [8]. Aamot et al. found significantly lower attendance with home exercise HIIT (4 × 4 min at 85–95% HR_max_ with 3-min active recoveries at 70% HR_max_, twice a week for 12 weeks) when compared to supervised treadmill or group HIIT sessions (*p* < 0.05) [20]. Given our program implemented supervised exercise sessions, this may explain our high attendance rates. Interestingly, most studies have not reported on the compliance to HIIT protocols [7, 8, 19, 27]. One study by Kim and colleagues reported that cardiac patients spent 86% of their exercise sessions within the target HR ranges with HIIT [21]. We similarly observed that most patients were able to meet or exceed the prescribed target HR for the HI (80%) and LO (84%) intervals, indicating that cardiac patients were able to comply with HIIT. Our qualitative data revealed that some patients found the location (i.e. an inconvenient/long distance) of the classes reduced their attendance. This is consistent with previous findings showing that when CR offerings are not easily accessible or convenient, it may be a barrier to cardiac patient participation [28, 29]. Given our high attendance, other community exercise programs may consider implementing HIIT for those with CVD.

Practitioners may be hesitant to prescribe HIIT in CR as high-intensity exercise acutely increases the risk of myocardial infarctions and sudden cardiac death, particularly in sedentary individuals [30]. Interestingly, we found that 27% of individuals exceeded the HI target HR range and very few adverse events occurred (3 out of 2224 training sessions) with HIIT. Our results are consistent with previous work indicating that adverse events are rare with HIIT in cardiac patients. A recent systematic review (*n* = 23 studies) showed that major cardiovascular events were rare when implementing HIIT in adults with coronary artery disease and heart failure with only one major cardiovascular event for 17,083 training sessions [14]. Vasovagal syncope is occurs more frequently within a CR setting due to the cardiovascular complications in this patient group [31]. In response to the vasovagal episodes which occurred following the HI work bouts at the start of the program, we implemented a step-by-step reduction in the exercise intensity following the HI intervals. This was to ensure patients had a more gradual reduction in HR to avoid future events [31]. No further vasovagal syncope episodes were reported. Our study supports previous work showing that HIIT is safe in a CR setting [14, 15] and the importance of a progressive reduction in HR following a HIIT session. Practitioners involved in CR should be reassured that the risk of an adverse event is small in cardiac patients. Our qualitative data showed that supervision during the HIIT classes helped patients feel safe and understand their physical abilities with exercise. Cardiac patients may not be aware of the low risk associated with HIIT and major cardiovascular events.

There is limited evidence investigating the patient experience with HIIT programs. Keteyian and colleagues implemented a similar HIIT program in a CR setting and found that patients reported a mean RPE of 15 and 12 for the HI and LO intervals, respectively [27]. We observed similar mean reported RPE during the HI (14 ± 1 points, “somewhat hard”) and LO (10 ± 2 points “very light”) intervals; these RPE scores were lower than the encouraged RPE ranges for the HR target ranges prescribed. A survey of 1273 cardiac patients found that a barrier for patients attending CR was perceiving exercise to be tiring or painful [32]. The integration of recovery periods in HIIT serves to reduce the fatigue and discomfort experienced by patients during exercise. This may explain why patients in our study have a high attendance and satisfaction. To our knowledge, this is the first study to examine cardiac patient perception regarding HIIT intensity difficulty; whether the program was challenging and satisfying for patients; and, the perceived patient safety of the program. Despite most patients finding the intensity of HIIT difficult and the classes challenging, all patients reported that they were satisfied with HIIT. Importantly, most patients thought HIIT was safe to perform and increased their confidence in their ability to exercise. A common barrier to participating in CR is low self-efficacy [28]. Given the individualized care and feedback that is often received in a supervised exercise program, patients can learn what their physical abilities are when exercising at higher intensities and improve their self-efficacy. Further, the patient’s experience with an exercise program is vital for predicting attendance [33]. It is important to note, providing RPE ranges alone may not achieve the target HR ranges needed for HIIT. Additional HR monitoring methods (i.e. chest straps, exercise machine sensors etc.) should be offered in combination with RPE when implementing HIIT to ensure patients are exercising at an appropriate intensity. Our study highlights that HIIT is well-received and an appealing exercise offering for cardiac patients which appears to lead to a positive patient experience.

The secondary aim of our study was to determine if there were sex differences in feasibility outcomes. While we did not observe any sex differences for most parameters, we found that significantly more women dropped-out of the HIIT program than men. This finding is consistent with a large study in 1088 women and 4833 men with coronary artery disease who were enrolled in CR which found women to withdraw from CR more often than men [34]. Further, we observed that more men were taking prescribed medications and had undergone an invasive procedure (coronary artery bypass grafting surgery) than women. These findings are unsurprising as men tend to receive more aggressive treatment for CVD than women [35–38]. For instance, men receive more cardiac catherizations (15.4% women, 27.3% men, *p* < 0.001) or coronary artery bypass graft surgeries (5.9% women, 12.7% men, p < 0.001) than women, despite women having greater functional disability with angina than men [37]. Given that men are more likely to receive medications and surgical interventions for CVD, they may be more informed about their medical condition and understand the importance of attending CR. This may leave women with a lack of knowledge regarding the severity and management of CVD [39], which may influence their participation in CR programs. Our study reinforces the findings of previous work showing that there is a need to understand how to attract and improve the retention of women in CR.

There are limitations that warrant mention. While this is the first study examining the feasibility of HIIT in cardiac patients, a retrospective analysis limits the ability to inform study design. For instance, we do not have the data to examine if patients complied to the duration of the HI and LO intervals. We did not download HR data from the HR monitors or the exercise machine HR sensors which may limit the accuracy of the data. Patients were instructed to report their typical HR for the HI and LO intervals, which may introduce response bias in the HR recordings. However, this provides “real world” data as patients are routinely asked to monitor and record their HR and or RPE in CR. This was also a single centre trial, which may limit the generalizability of our results across other CR settings. Other aspects of the patient’s experience should be explored such as patient confidence and self-efficacy to more thoroughly examine if HIIT is feasible for this patient group. Similar to previous work [8, 18–20, 22, 27], significantly more men participated in this study than women; the results from our sex analysis should, therefore, be interpreted with caution. Knowing the feasibility of an exercise provides valuable insight for practitioners who may wish to offer HIIT for their cardiac patients but are unsure of the possible challenges with instructing HIIT in this population.

## Conclusion

HIIT is a well-received, safe and feasible exercise program within CR settings. All patients were satisfied with HIIT and most patients found the program to be challenging and improved their confidence to exercise. Sex-based analysis revealed that women were more likely to drop-out of HIIT, however, there were no sex differences for all other feasibility outcomes (i.e. attendance, compliance, patient experience, safety). HIIT is a suitable exercise modality for CR.

## Data Availability

The datasets during and/or analysed during the current study available from the corresponding author on reasonable request.

## Abbreviations

ACE: Angiotensin-converting enzyme inhibitor
BMI: Body mass index
CPET: Cardiopulmonary exercise test
CR: Cardiac rehabilitation
CVD: Cardiovascular disease
HI: High-intensity interval
HIIT: High-intensity interval training
HR: Heart rate
HR_max_: Maximum heart rate
HR_peak_: Peak heart rate
MET: Metabolic equivalent
LO: Lower-intensity interval
UOHI: University of Ottawa Heart Institute
V̇O_2peak_: Peak exercise capacity
